# Advances in Topical Therapies for Clinically Relevant and Prevalent Forms of Alopecia

**DOI:** 10.3390/life14121577

**Published:** 2024-12-01

**Authors:** Aarushi K. Parikh, Isabella J. Tan, Sydney M. Wolfe, Bernard A. Cohen

**Affiliations:** 1 Rutgers Robert Wood Johnson Medical School, 125 Paterson St, New Brunswick, NJ 08901, USA; sw1175@rwjms.rutgers.edu; 2 Department of Dermatology, The Johns Hopkins Hospital, Baltimore, MD 21287, USA; bcohena@jhmi.edu

**Keywords:** alopecia, topical treatments, minoxidil, corticosteroids, Janus kinase (JAK) inhibitors, hair regrowth

## Abstract

Alopecia encompasses diverse conditions that vary by etiology, progression, and clinical presentation, including androgenetic alopecia, alopecia areata, telogen effluvium, and scarring alopecias such as lichen planopilaris and central centrifugal cicatricial alopecia. Managing these conditions requires tailored therapeutic approaches, with topical treatments emerging as effective first-line interventions. This literature review examines topical therapies across alopecia types, assessing mechanisms of action, clinical efficacy, and safety profiles to guide evidence-based clinical practice. Methods involved a comprehensive search across PubMed, SCOPUS, and Web of Science databases, focusing on clinical research published within the past five years. Articles were screened based on relevance to alopecia management, excluding abstracts, non-English studies, and ongoing research. Topics covered include commonly used agents such as minoxidil, corticosteroids, and emerging options like Janus kinase (JAK) inhibitors. Topicals for trichotillomania, such as capsaicin and numbing creams, are highlighted for their behavioral conditioning potential, while treatments like minoxidil and adenosine are explored for telogen effluvium. Findings indicate that topicals provide symptom relief, promote hair regrowth, and often serve as adjuncts to systemic therapies. Minoxidil and corticosteroids demonstrate efficacy in multiple alopecia types, while JAK inhibitors show promise in alopecia areata. This review underscores the value of topical treatments in alopecia management and highlights areas for future research, advocating for individualized approaches to enhance therapeutic outcomes in patients experiencing hair loss.

## 1. Introduction

Alopecia, a broad term for hair loss, encompasses a variety of types that affect individuals differently based on etiology, progression, and clinical presentation. Common forms include androgenetic alopecia (AGA), alopecia areata (AA), telogen effluvium (TE), and scarring alopecias such as lichen planopilaris (LPP) and central centrifugal cicatricial alopecia (CCCA), with other causes like discoid lupus erythematosus, scleroderma, and folliculitis decalvans, although these are less common primary scalp issues. Each type is characterized by distinct underlying mechanisms. AGA is primarily driven by genetic predisposition and hormonal factors, particularly androgens, which lead to gradual hair thinning. AA is an autoimmune disorder where the immune system mistakenly targets hair follicles, leading to patchy hair loss. TE is typically triggered by stress or physiological changes like illness, medication, or hormonal fluctuations, causing widespread shedding. Scarring alopecias, such as LPP and CCCA, are characterized by inflammatory processes that permanently damage hair follicles, replacing them with scar tissue.

The classification of alopecia is generally divided into non-scarring and scarring types, each of which can be further subdivided based on their underlying pathophysiological mechanisms. Non-scarring alopecias, like AGA, AA, and TE, typically result in temporary or reversible hair loss, whereas scarring alopecias lead to irreversible follicular destruction. The etiologies of alopecia are highly heterogeneous and involve a range of factors: genetic, hormonal, autoimmune, inflammatory, and environmental. For example, AGA is linked to a genetic predisposition to sensitivity to androgens, whereas AA etiology is largely autoimmune, with genetic and environmental triggers also playing a role. TE is often stress-related or triggered by metabolic disruptions while scarring alopecias like LPP and CCCA are driven by chronic inflammation that leads to follicular destruction. Understanding these diverse causes is critical for accurate diagnosis and treatment, as management strategies must be tailored to the specific underlying mechanisms of each type of alopecia.

Topical treatments have gained prominence as non-invasive, first-line options for managing various forms of alopecia, with a wide range of innovative treatments. Minoxidil, an over-the-counter vasodilator, is commonly used for androgenetic alopecia (AGA) and promotes hair regrowth by improving blood flow to hair follicles. Minoxidil, an over-the-counter vasodilator, is commonly used for AGA. Its mechanism of action is multifaceted, involving the stimulation of hair follicle activity through increased vascular endothelial growth factor (VEGF) expression, prolongation of the anagen phase, and potassium channel opening, which improves follicular blood flow and nutrient delivery. Corticosteroids, often prescribed for autoimmune forms like alopecia areata (AA), reduce inflammation around hair follicles and suppress immune responses, promoting regrowth in affected areas. Calcineurin inhibitors such as tacrolimus and pimecrolimus are used as steroid-sparing alternatives in AA, blocking immune cell activation to prevent follicular damage. More recently, Janus kinase (JAK) inhibitors like tofacitinib have shown promise in treating alopecia areata by targeting the enzymes involved in the inflammatory process, offering a novel approach for immune-mediated hair loss. Topical immunotherapy and phototherapy are also treatment options for alopecia areata. These diverse treatments offer varying degrees of efficacy depending on the underlying cause of hair loss, making it important to select the appropriate therapy for each patient. The efficacy and safety profiles of these topical agents vary, influenced by factors such as the type of alopecia, patient demographics, and underlying pathophysiology.

This literature review aims to provide a comprehensive overview of topical treatments for different types of alopecia that have clear diagnostic criteria, prevalent treatment approaches, and ongoing clinical relevance. We sought to evaluate their mechanisms of action and clinical efficacy in order to inform clinical practice and guide future investigations into optimizing treatment strategies for patients experiencing hair loss.

This review distinguishes itself from the existing literature by providing a comprehensive analysis of topical therapies across multiple types of alopecia, while prior reviews often focus on single conditions or systemic therapies. This review uniquely addresses underrepresented conditions, integrates novel and emerging treatments, and discusses comparative efficacy insights and behavioral approaches to advance the understanding of precision topical therapies.

## 2. Methods

### 2.1. Research Question

What are the mechanisms of action, clinical efficacy, and safety profiles of topical treatments for the various types of alopecia, and how can these therapies be optimized to improve patient outcomes?

### 2.2. Search Strategy

This literature review was conducted utilizing Pubmed (Medline), SCOPUS, and Web of Science databases. Using the NLM Medical Subject Heading (MeSH) to determine the best selection of potential search terms, the following strings were derived and used: (“topical”) AND (“Trichotillomania” OR “Telogen effluvium” OR “Anagen effluvium” OR “Androgenetic alopecia” OR “Alopecia areata” OR “Alopecia totalis” OR “Alopecia universalis” OR “traction alopecia” OR “Central Centrifugal Cicatricial Alopecia”). Articles included were clinical research studies (randomized controlled trials, cohort studies, retrospective studies, case reports, case series), published within the last 5 years (January 2019–August 2024), and pertained to clinical manifestations, treatments, and ongoing research of common types of alopecia. Secondary sources, such as other reviews, were considered and explored to supplement any missing information. Abstracts, articles lacking full text, studies still in progress, and articles not in English availability were excluded. Full-text appraisal was performed by two reviewers (AKP, IJT), with articles analyzed for relevance and proper data reporting. Additionally, in cases where relevant articles were identified outside the original search terms, they were incorporated into the review after careful evaluation to ensure they met the established inclusion criteria.

## 3. Results and Discussion

The results of this literature review highlight the mechanisms of action, clinical efficacy, and safety profiles of current and emerging topical therapies for the treatment of various types of alopecia. These results synthesize findings from recent studies, emphasizing therapeutic potential, limitations, and future directions for optimizing treatment strategies tailored to patients’ needs.

### 3.1. Trichotillomania

Trichotillomania (TTM) is a psychological disorder characterized by the compulsive urge to pull out one’s own hair. This condition is classified as a type of obsessive-compulsive and related disorder and can affect various areas of the body, including the scalp, eyebrows, eyelashes, and other body parts. First-line therapy for TTM is psychiatric care, such as cognitive behavioral therapy (CBT) [1]. However, topicals that impact the sensations on the scalp have been utilized in treatment and management, especially when TMM patients fail to completely respond to first-line treatment and/or selective serotonin reuptake inhibitors (SSRIs), leaving few other treatment options for patients of all ages (Table 1) [2]. Numbing creams such as benzocaine have been used to treat the aftermath of hair-pulling. These creams provide temporary relief from pain or discomfort in the affected areas, helping to soothe irritated skin and potentially reducing the urge to pull by alleviating discomfort. On the other spectrum, pain-enhancing creams with capsaicin have also been used to disrupt the hair-pulling that is characteristic of the condition [3]. Capsaicin, the active compound in chili peppers, creates a burning sensation that can act as a deterrent to hair-pulling by making the act uncomfortable or painful. This method leverages the principle of aversive conditioning, where the discomfort caused by the capsaicin can help reduce the urge to pull hair. As behavioral support is a predominant aspect of TTM treatment, such topical treatment addresses this need.

N-acetylcysteine (NAC) has also demonstrated significant reductions in TTM symptoms. The mechanism of action of NAC in TTM is believed to involve its effect on neurotransmitter systems, particularly through the reduction of glutamate levels in the nucleus accumbens [4]. NAC is known for its role as a precursor to glutathione, a powerful antioxidant, and for modulating glutamate levels, an excitatory neurotransmitter. In TTM, excessive glutamate activity in the nucleus accumbens—a key area of the brain involved in reward and impulse control—can contribute to compulsive behaviors. By decreasing glutamate levels, NAC may help rebalance neurotransmitter activity, thereby reducing the intensity of compulsive urges and aiding in the management of TTM. This neurochemical modulation is thought to complement behavioral therapies and other treatments in addressing the disorder. Clinical trials also demonstrate promise at relieving compulsive behavior, with a double-blind placebo-controlled trial using 1200–2400 mg/day NAC showing significant reductions in hair pulling based on the Massachusetts General Hospital Hair Pulling Scale (*p* < 0.001) and the Psychiatric Institute Trichotillomania Scale (*p* = 0.001) compared to placebo at 12 weeks [5].

Other methods that have shown varying effectiveness in the literature involve mild shampoos and topical steroids, which help reduce irritation and alleviate inflammation and discomfort from pulling [6,7,8]. While these approaches may provide some relief, they are most effective when integrated into a comprehensive treatment plan addressing both physical and psychological aspects of the condition.

As a whole, additionally, the routine of applying a topical treatment can serve as a behavioral anchor, promoting mindfulness and distraction from the urge to pull. By integrating these creams into a comprehensive treatment plan, individuals may find it easier to manage their symptoms and foster healthier coping mechanisms. Hence, incorporating topical treatments with behavioral support features can enhance the overall management of trichotillomania, offering individuals a practical and effective tool to address both the physical and psychological aspects of their condition.

### 3.2. Telogen Effluvium

Telogen effluvium (TE) is a form of non-scarring alopecia characterized by excessive shedding of telogen hairs beyond the normal range, typically triggered by a variety of precipitating factors, including acute illness, surgery, emotional stress, hormonal changes (e.g., postpartum), nutritional deficiencies, and certain medications [9]. This condition affects both men and women, with a prevalence of up to 30% among individuals experiencing hair loss [9].

The pathophysiology of TE involves a disruption in the hair growth cycle, shifting an increased number of follicles into the telogen (resting) phase [10]. Normally, approximately 10–15% of scalp hairs are in telogen phase at any given time, but in TE, this percentage can rise to 30% or higher [10]. This results in noticeable hair shedding approximately 2–3 months after the inciting trigger, as affected hairs are prematurely shed before entering the anagen (growth) phase [10].

Clinically, TE presents as diffuse hair thinning rather than focal bald patches, which distinguishes it from other forms of alopecia [11]. Diagnosis often involves a thorough medical history to identify potential triggers and rule out other causes of hair loss through physical examination and laboratory tests, including pull tests and trichoscopy [11].

**Figure 1:**
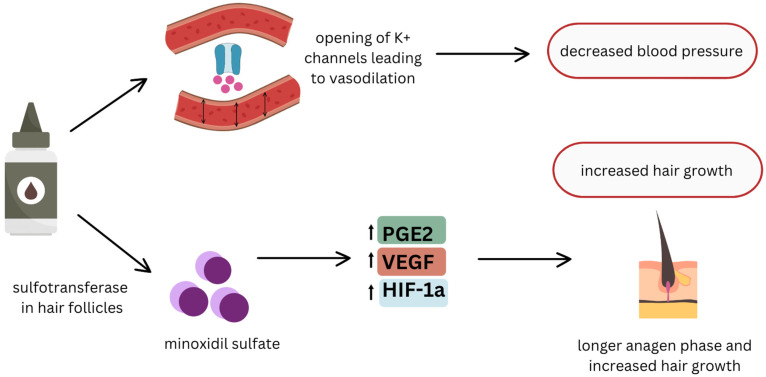
Mechanism of Action of Topical Minoxidil in Hair Growth Promotion.

Management of TE primarily focuses on addressing the underlying trigger, as the condition is usually self-limiting and resolves within 6–12 months once the inciting factor is removed [11]. However, topical therapies have been explored to potentially accelerate recovery or mitigate hair loss severity. Recent advances in topical therapies for alopecias, including TE, have highlighted promising results with ingredients such as minoxidil and other growth factors that stimulate hair follicle activity and shorten the telogen phase (Figure 1) [12].

Studies have shown that topical minoxidil, a vasodilator originally used for hypertension, can effectively promote hair growth by extending the anagen phase and stimulating follicular proliferation [12]. Topical minoxidil, including 2% and 5% formulations (including foam), is FDA-approved for androgenetic alopecia in both men and women. One review highlighted data showing the efficacy of topical minoxidil in promoting hair regrowth in frontotemporal and vertex areas, with comparable effectiveness between the 5% and 2% solutions [12]. It has been clinically demonstrated to reduce hair shedding and improve hair density in patients with TE [12]. The study also discussed findings indicating that oral minoxidil (5 mg/day) may be more effective than topical formulations after 6 months in male AGA and that low-dose oral minoxidil (0.5–5 mg/day) was safe and effective for female pattern hair loss and chronic telogen effluvium. Sublingual minoxidil was also noted as potentially safe and effective for TE [12].

Additionally, formulations of topical minoxidil (5%) enriched with adenosine (0.75%), a purine nucleoside known for its role in cellular energy metabolism, have shown efficacy in enhancing hair growth and reducing shedding by promoting anagen phase duration [13].

Furthermore, research into topical corticosteroids has indicated their potential role in managing inflammation associated with TE, thereby modulating the hair cycle and reducing hair loss severity [10]. Topical corticosteroids work by suppressing immune responses that may disrupt follicular activity during the telogen phase [10]. One review identified potential effective treatments including corticosteroids and novel therapies like CNPDA (caffeine, niacinamide, panthenol, dimethicone, and an acrylate polymer), which was shown to increase the cross-sectional area of scalp hair fibers by 10%, thereby targeting symptom relief and hair regrowth promotion in TE [10].

### 3.3. Anagen Effluvium

Anagen effluvium (AE) is a type of alopecia characterized by the sudden cessation of hair growth during the anagen (growth) phase of the hair cycle. Unlike telogen effluvium, which primarily involves the shedding of telogen-phase hairs, AE results in abrupt and widespread hair loss due to damage to actively growing hair follicles [14]. This condition is commonly associated with exposure to cytotoxic agents such as chemotherapy drugs, which disrupt rapidly dividing cells, including hair matrix cells [14].

The pathophysiology of AE involves direct toxicity to the hair matrix cells responsible for hair shaft formation during the anagen phase [14]. Systemic chemotherapeutic agents target rapidly dividing cells and inadvertently affect hair follicles in their active growth phase. This results in the sudden onset of diffuse hair shedding within weeks of initiating chemotherapy.

Clinically, AE presents as rapid and severe hair loss, often affecting the scalp but occasionally involving other body hair as well. The pattern of hair loss is typically diffuse and can be emotionally distressing for patients undergoing cancer treatment.

Diagnosis of AE is straightforward in the context of chemotherapy administration and can be confirmed through clinical history and physical examination [15]. A scalp biopsy may be performed in some cases to confirm the diagnosis and rule out other causes of hair loss [15].

Management of AE primarily revolves around supportive care and mitigating factors contributing to hair loss, as the condition is usually reversible once chemotherapy is completed and hair follicles recover [15]. Topical therapies have been explored to potentially enhance hair regrowth and improve cosmetic outcomes during and after chemotherapy-induced hair loss [15].

Recent advances in topical therapies for alopecias, including AE, have focused on strategies to support hair follicle recovery and stimulate regrowth. Minoxidil, a vasodilator known for its hair growth-promoting effects, has shown promise in clinical studies by promoting the transition of hair follicles from the telogen to the anagen phase and increasing hair shaft diameter [12]. This mechanism may accelerate the recovery of hair growth following chemotherapy-induced AE.

Additionally, topical formulations containing growth factors such as bimatoprost, a prostaglandin analog, have demonstrated efficacy in enhancing eyelash growth and may have potential applications in promoting hair regrowth in AE [16]. Topical prostaglandin analogs significantly improved hair length and density compared to placebo across six RCTs with seven datasets (SMD = 1.42, 95% CI: 0.66–2.18, *p* = 0.0003), with one study showing a 39.1% increase in hair growth versus 35.6% in controls and another reporting 30.31 mm growth versus 2.59 mm in controls. While there was a slight increase in mild adverse events like local irritation (*p* = 0.07, I^2^ = 54%), the safety profile was generally acceptable. Approximately 16 these growth factors act on follicular stem cells to stimulate proliferation and differentiation, thereby supporting the regeneration of hair follicles damaged by chemotherapy [16]. Topical prostaglandin analogs, as studied in six randomized controlled trials, were found to significantly improve hair length and density compared to placebo (*p* < 0.001), with no significant difference in adverse events observed between the experimental and control groups [16].

Moreover, novel formulations enriched with botanical extracts and peptides have shown promising results in preclinical studies by exerting protective effects on hair follicles and enhancing hair shaft integrity [17]. Various phytochemicals, including epigallocatechin gallate (EGCG), caffeine, capsaicin, procyanidin, onion juice, pumpkin seed oil, rosemary oil, saw palmetto, red ginseng extract, curcumin, garlic gel, amino acids, marine proteins, melatonin, vitamins, and zinc, have demonstrated hair growth-stimulating properties [17]. Recent research has focused on herbal-based nanomedicine for hair health, while several mechanisms underlying the hair growth-promoting effects of phytochemicals have been proposed and substantiated [17]. These ingredients may mitigate the cytotoxic effects of chemotherapy on hair follicles and promote faster recovery of hair growth [17].

### 3.4. Androgenetic Alopecia

Androgenetic alopecia (AGA), commonly known as male or female pattern baldness, is a prevalent condition characterized by hair thinning and loss due to genetic and hormonal factors. The condition is largely hereditary, with specific genes related to androgen receptors implicated in its development, suggesting a strong genetic component. At the core of AGA is the role of dihydrotestosterone (DHT), a derivative of testosterone, which is produced through the action of the enzyme 5-alpha-reductase. In individuals with a genetic predisposition, hair follicles become hypersensitive to DHT, leading to follicular miniaturization, where hair follicles shrink, producing thinner and weaker hairs. This miniaturization disrupts the normal hair growth cycle by shortening the anagen (growth) phase, increasing the number of hairs entering the telogen (resting) phase, and resulting in increased shedding. Chronic low-grade inflammation through variables such as diet may exacerbate the condition, as factors such as oxidative stress and cytokines contribute to follicle damage [18]. While various treatments exist, topical therapies have gained particular attention for their ease of use and direct application to affected areas.

As with many hair loss disorders, minoxidil is the most well-established topical treatment for AGA in both males and females [19]. Available over-the-counter in various formulations, including liquid and foam, minoxidil is applied directly to the scalp.

Though primarily known as an oral medication, finasteride has been studied in topical formulations (0.1% finasteride, 3% minoxidil) for its potential to reduce hair loss in male AGA [19]. By inhibiting the enzyme 5-alpha-reductase, finasteride decreases the conversion of testosterone to DHT. Off-label use of topical finasteride has shown similar efficacy to oral finasteride with potentially fewer systemic side effects. However, it is not yet FDA-approved for topical use in AGA [20].

Ketoconazole, an antifungal medication, is often used in shampoo form as a treatment for dandruff and seborrheic dermatitis. However, its role in AGA has garnered interest due to its anti-androgenic properties. Studies report increased hair shaft diameter and improved overall scalp health with 1–2% ketoconazole formulations, possibly contributing to enhanced hair density [21]. While these findings are promising, further research is needed to establish its effectiveness and clarify its role as a standalone treatment for AGA.

Non-pharmaceutical topics have also shown efficacy in treating AGA. Some studies have shown that caffeine-based topical solutions may be non-inferior to minoxidil 5% in treating male pattern hair loss [22]. These products may work by stimulating hair follicles and prolonging the anagen phase of hair growth through migration of human hair follicle dermal papilla cells and activation of various genetic hair growth pathways [23]. Topical preparations of *Serenoa repens* (Saw palmetto) extract have shown some efficacy in increasing hair count and density while leading to reduced hair fall as well [24,25]. The inclusion of such ingredients in hair supplements has yet to be studied but holds potential in AGA therapies.

### 3.5. Alopecia Areata (Including Alopecia Totalis and Alopecia Universalis)

Alopecia areata (AA) is a chronic autoimmune disease characterized by a T-cell attack on hair follicles, leading to non-scarring hair loss. Precipitating factors in the development of AA include stress, trauma, infection, atopic background, and genetic predispositions that lead to a dysregulated immune system [26]. AA presents in varying degrees of severity, with patchy hair loss (mild-moderate AA), complete scalp hair loss (alopecia totalis), or hair loss across the entire body (alopecia universalis) [26]. The “exclamation mark hairs” that often arise around the periphery of hair loss patches reflect the follicular inflammation and damage associated with AA, particularly during active disease phases.

Management of AA is highly individualized, depending on the patient’s age, extent of hair loss, and chronicity of the disease. Various agents with distinct mechanisms of action may be used in the treatment of AA, and there is currently no consensus on optimal treatment plans for long-term management of AA [26]. The use of topical agents in AA treatment is favorable due to the limited or local side effects compared to systemic therapies. For mild or patchy AA, topical corticosteroids are considered first-line treatments [27]. Corticosteroids function to suppress the local immune responses that drive follicular inflammation, helping to promote hair regrowth. Corticosteroids have been shown to be a safe, reliable, and frequently used treatment in the management of mild-moderate AA [28]. As topical corticosteroids can induce side effects including telangiectasias, skin atrophy, and striae, they are only approved for short-term use, indicating a need for other topical treatment options that are effective with minimal side effects for long-term management of AA [29,30]. Other immunosuppressive agents such as methotrexate, cyclosporine, and azathioprine have been used in the treatment of AA; however, these agents lack sufficient RCT evidence to support their use and require extensive patient follow-up due to the risks of severe immunosuppression [30].

In cases of severe AA or resistance to corticosteroids, contact immunotherapy with squaric acid dibutylester (SADBE) and diphenylcyclopropenone (DPCP) is often used [31,32]. These treatments induce mild dermatitis to divert immune cells away from the hair follicles and reduce the number of T-cells in the peribulbar area [33]. Clinical studies have shown that topical immunotherapy with SADBE can achieve partial or complete hair regrowth in approximately 57% of AA patients [33]. Another study evaluated treatment with DPCP in AT and AU patients, demonstrating >75% hair regrowth in 33.3% of these patients [32]. Of note, the combination of DPCP and 0.5% anthralin was found not to be superior to DPCP alone for the treatment of chronic extensive AA, and there were significant side effects, including excessive dermatitis and hyperpigmentation [33]. All in all, contact immunotherapy is commonly used in the treatment of refractory or severe AA, as agents have shown some benefit for hair growth in these patient populations.

Topical calcipotriol has been shown to be reasonably safe and effective for treating AA, with limited and reversible side effects [34]. Calcipotriol is a vitamin D analog that acts as a potent immunomodulatory agent that binds to specific vitamin D receptors (VDR) in keratinocytes to maintain hair follicle growth and epidermal differentiation [34]. A recent RCT found that treatment with calcipotriol for 3 months significantly improved SALT scores in patchy AA cases compared to placebo, indicating its potential as an effective topical therapy for AA [35]. A study investigating the efficacy and safety of treatment with calcipotriol (calcipotriol 0.005% ointment) versus treatment with the corticosteroid clobetasol (topical clobetasol 0.05% formulation) in a series of 35 patients with scalp AA showed that patches treated with calcipotriol ointment had greater and faster response rates than did those treated with topical clobetasol, although the differences were not statistically significant [34]. A randomized control trial with 60 chronic (>1 year) AA patients (SALT score < 25%) randomized to treatment with topical calcineurin inhibitor (0.003% tacrolimus), topical potent steroid combined with vitamin D analog (Daivobet^®^), or topical superpotent steroid (Dermovate^®^) showed that vitamin D analogs combined with potent steroids were shown to be more favorable than superpotent steroids due to fewer side effects and comparable efficacy [36]. Tacrolimus yielded the least improvement despite its immunosuppressive mechanism of action, indicating that further research is required to establish the utility of calcineurin inhibitors such as tacrolimus and pimecrolimus in the treatment of AA [36].

Minoxidil, a topical vasodilator commonly used in the treatment of AGA, is being increasingly used in the treatment of AA and should ideally be used in combination with other therapies. Minoxidil promotes hair regrowth by prolonging the anagen phase of hair follicles and increasing blood supply to the scalp. A recent network meta-analysis found a 67.1% likelihood of response in mild AA and a 55.5% likelihood of response in severe AA (including AT and AU) with topical minoxidil treatment alone [37]. While topical minoxidil has been evaluated in many RCTs, its treatment effect was lower than topical corticosteroids, as established by a systematic review with network analysis aimed at ranking treatments [38]. Furthermore, minoxidil plus topical corticosteroids are effective but not superior to topical corticosteroids alone [38].

Recent studies have explored the potential of topical JAK inhibitors, such as tofacitinib and ruxolitinib, in the treatment of AA. JAK inhibitors block pathways critical to cytokine receptor signaling pathways and subsequent autoimmune responses, thereby reducing hair follicle inflammation in AA [39]. Case studies and case series evaluating the efficacy of topical JAK inhibitors (5 mg, twice per day) have shown benefits in hair growth; however, these agents are still under investigation as there are limited randomized controlled trials to yield conclusive evidence [30,40].

Overall, topical corticosteroids remain the cornerstone of AA treatment, but other topical therapies such as calcipotriol and minoxidil show potential for use in the long-term management of AA. While topical immunotherapies are effective in refractory cases, their varying clinical efficacy and side effects necessitate further trials to optimize treatment strategies. These findings highlight the need for more robust clinical trials to compare efficacy and establish clear treatment protocols for AA patients.

### 3.6. Central Centrifugal Cicatricial Alopecia

Central Centrifugal Cicatricial Alopecia (CCCA) presents a significant therapeutic challenge due to its scarring alopecia nature and the limited efficacy of current treatments, which primarily focus on inflammation management rather than hair regrowth promotion. CCCA predominantly affects individuals of African descent, particularly women, and is characterized by progressive hair loss starting from the central scalp and spreading outward [41]. The condition is associated with both genetic predisposition and external factors, such as traumatic hairstyling practices [41]. Histologically, CCCA is marked by follicular inflammation, perifollicular fibrosis, and irreversible scarring, leading to permanent hair loss [41].

Topical corticosteroids represent a mainstay in treatment, offering anti-inflammatory benefits, but their prolonged use raises concerns about cutaneous side effects and systemic absorption [41]. Treatment goals for CCCA aim to encourage hair regrowth and halt disease progression, although regrowth from permanently damaged follicles is not feasible. Topical steroids or intralesional triamcinolone acetonide are commonly employed as first-line treatments, with lower concentrations of topical steroids reducing the risk of hypopigmentation in darker skin types [41]. Other treatment options include antibiotics including doxycycline over 2–6 months, systemic therapies such as mycophenolate mofetil and corticosteroids for severe cases, and potential benefits from minoxidil and tacrolimus, with emphasis on minimizing hair grooming and using mild shampoos for symptomatic relief [41].

Emerging therapies such as topical minoxidil have shown promise by prolonging the anagen phase of hair growth and enhancing follicular diameter, potentially supporting healthier hair cycles and mitigating inflammation in CCCA [41]. Furthermore, calcineurin inhibitors such as tacrolimus and pimecrolimus are being investigated for their ability to suppress inflammatory T_H_1 cytokines within hair follicles, providing alternatives to corticosteroids [42]. These agents have been utilized to hinder disease progression when applied topically or via intralesional injection [42]. However, further well-designed randomized controlled trials are necessary to establish their optimal efficacy and safety profiles in treating CCCA.

Novel topical formulations enriched with growth factors, peptides, and botanical extracts also aim to improve the follicular microenvironment and enhance hair shaft integrity, potentially reversing early-stage scarring and supporting hair regrowth [41]. One case series explored the therapeutic efficacy of a new botanical formulation (Gashee) in four African American women with treatment-resistant CCCA [43]. The topical formulation, which includes multiple phytoactive ingredients and is applied with 1 mL per palm-sized area, led to cessation of scalp pruritus within 2 weeks and significant hair regrowth within 2 months of treatment initiation [43]. Patient satisfaction was high, and no adverse effects were reported with the topical and oral formulations used alone or in combination over treatment periods ranging from 8 weeks to 1 year [43].

Despite these advancements, optimizing treatment regimens, enhancing therapeutic outcomes, and minimizing adverse effects remain critical challenges. Future research efforts should focus on conducting large-scale clinical trials to establish comparative effectiveness, exploring combination therapies targeting multiple aspects of CCCA pathogenesis, and identifying biomarkers predictive of treatment response. Advances in genetic and molecular understanding may reveal new targets for topical interventions, thereby expanding therapeutic options for CCCA management and improving overall patient outcomes.

## 4. Conclusions

Advancements in topical therapies for alopecias offer promising treatment options across a range of hair loss disorders, including androgenetic alopecia, alopecia areata, telogen effluvium, and anagen effluvium. Newer light and laser therapies have been explored in recent years for treating various forms of alopecia. One systematic review of 58 studies found that treatments like excimer laser (308 nm) and low-level light therapy (LLLT) significantly improved hair density and diameter in alopecia areata (*n* = 261) and androgenetic alopecia (*n* = 919); erbium-glass and thulium lasers also showed notable efficacy in androgenetic alopecia (*n* = 172). However, LLLT did not significantly improve outcomes in telogen effluvium (*n* = 1 study), and narrow-band UVB was less effective compared to corticosteroids for alopecia areata (SALT score reduced from 3.97 to 2.33, *p* < 0.001). Common side effects included scalp tenderness, erythema, and pruritus.

The reviewed literature highlights the growing efficacy of agents such as minoxidil, corticosteroids, and novel formulations, including JAK inhibitors and phytochemicals. These topical treatments, valued for their accessibility and minimal systemic side effects, have broadened the therapeutic landscape for alopecia patients. To address the variety of alopecias, we recognize the importance of optimal management for both non-scarring and scarring types. Common non-scarring alopecias include androgenetic alopecia, alopecia areata, and telogen effluvium, each requiring distinct therapeutic strategies based on underlying causes and progression. Scarring alopecias, such as lichen planopilaris and central centrifugal cicatricial alopecia, necessitate more targeted treatments to prevent further follicular damage and preserve hair density. These forms of alopecia highlight the need for personalized, evidence-based approaches to management, incorporating both topical therapies and, in some cases, systemic interventions.

The study of topical treatments for hair loss is essential for advancing effective management strategies. These therapies offer greater accessibility and targeted action, minimizing systemic side effects and allowing for localized effects. By exploring the diverse mechanisms of action, researchers can develop more effective therapies that enhance patient adherence and outcomes. Innovations in formulations and delivery systems also pave the way for personalized approaches tailored to individual needs and types of hair loss. Moreover, the preference for non-invasive options among patients underscores the importance of refining these treatments. As topical therapies often prove to be more cost-effective than systemic alternatives, they present significant benefits for both patients and healthcare systems. Continued research is essential to optimize formulations, enhance efficacy, and further personalize treatment strategies based on individual pathophysiology and alopecia type. Collaborative efforts among researchers, clinicians, and patients will foster innovation and lead to the development of comprehensive treatment protocols that address the diverse needs of those affected by hair loss.

## Figures and Tables

**Table 1:** Topical Treatments for Scalp and Hair Disorders.

Condition	Description	First-Line Treatments	Additional Treatments
Trichotillomania	Compulsive hair-pulling leads to hair loss.	Cognitive Behavioral Therapy (CBT), SSRIs	N-acetylcysteine (NAC) Topical numbing creams Capsaicin creams Mild shampoos Topical steroids
Telogen Effluvium	Temporary hair loss due to stress or illness results in excessive shedding.	Addressing underlying triggers, reassurance about the self-limiting nature.	Topical minoxidil (2% and 5%) Oral minoxidil Adenosine formulations Topical corticosteroids CNPDA
Anagen Effluvium	Sudden hair loss during the anagen phase, often due to chemotherapy.	Supportive care, monitoring hair regrowth post-chemotherapy.	Topical minoxidil Growth factors (e.g., bimatoprost) Botanical extracts and peptides
Androgenetic alopecia	Genetic and hormonal hair loss, is common in males and females.	Minoxidil	Finasteride (oral and topical) Ketoconazole shampoo Caffeine-based solutions Saw palmetto extract
Alopecia Areata	Autoimmune hair loss can vary from patchy to total loss.	Topical corticosteroids	Intralesional corticosteroids Contact immunotherapy (SADBE, DPCP) Calcipotriol Minoxidil Topical calcineurin inhibitors JAK inhibitors (e.g., tofacitinib, ruxolitinib) Immunosuppressants (methotrexate, cyclosporine, and azathioprine)
Central Centrifugal Cicatricial Alopecia	Scarring alopecia predominantly affects women of African descent, marked by hair loss from the center of the scalp outward.	- Topical corticosteroids - Intralesional triamcinolone acetonide	Antibiotics (e.g., doxycycline) Systemic therapies (mycophenolate mofetil, corticosteroids) Minoxidil Tacrolimus Mild shampoos Novel topical formulations (growth factors, peptides, botanical extracts) Calcineurin inhibitors (tacrolimus, pimecrolimus)

## Data Availability

The raw data supporting the conclusions of this article will be made available by the authors on request.
